# Cardiovascular risk evaluation using lipid profile and blood pressure among obese and non-obese individuals in India

**DOI:** 10.6026/973206300200723

**Published:** 2024-07-31

**Authors:** Rafae Taqiuddin, Hemaakshi Gupta, Zoha Sufian, Khaja Kamaluddin, Yasir Adil El Rashid Mohamed, Ariyan Khan, Hamza Abdulmunem Orfali, Nimerta Lohana, Mohamedelfatih musaab Ibrahim Mohamed, Mohammed Abdul Mateen

**Affiliations:** 1 Shadan Institute of Medical Sciences, Teaching Hospital and Research Centre, Hyderabad, India; 2 American University of the Carribean, Jordan Dr, Cupecoy, Sint Maarten; 3 Dr.VRK Womens Medical College, Hyderabad, India; 4 M.B.B.S., Deccan College of Medical Sciences, Hyderabad, India; 5 General physician., Military Medical Hospital, Omdurman, Khartoum, Sudan; 6 Deccan College of Medical Sciences, Hyderabad, India; 7 Faculty of Medicine, Al-Neelain University, Khartoum, Sudan; 8 Liaquat University of Medical and Health Sciences, Jamshoro, Sindh, Pakistan; 9 General Practitioner., National Ribat University, Khartoum, Sudan; 10 Shadan Institute of Medical Sciences Teaching Hospital and Research Centre, Hyderabad, India

**Keywords:** Lipid Profile, Obese and Non-Obese Individuals, Blood pressure, Body mass index

## Abstract

Cardiovascular diseases (CVDs) are a group of disorders that affect the heart and blood vessels. Identifying high-risk individuals is a primary goal of cardiovascular disease prevention. The aim is to examine risk factor on assessing lipid profiles and blood pressure, both in obese and non-obese individuals. This study took place over a year at a Tertiary Care Hospital, were investigated the lipid profile and blood pressure of obese and non-obese participants aged 30-60 years. The obese group had an average age of 43.2±6.3 years compared to 45.1±5.8 years in the non-obese group, indicating a slightly older population in the obese group. The non-obese group had an average total cholesterol level of 193.7 mg/dL, with a total cholesterol (TC) level of 209.3 mg/dL. When it came to LDL cholesterol, the obese group had a higher level of 137.4 mg/dL compared to the non-obese group with 121.3 mg/dL. Conversely, HDL cholesterol levels were lower in the obese group at 44.1 mg/dL than in the non-obese group at 49.1 mg/dL. Obesity is associated with lipid metabolism and hypertension disturbances, especially with effect on HDL-C reduction and TC, TG, LDL cholesterol to high level. Thus, lipid profile and blood pressure among obese and non-obese individuals help in cardiovascular risk evaluation.

## Background:

High blood pressure poses a significant risk to the cardiovascular health of middle-aged individuals in India. This condition is characterized by a systolic blood pressure of 140 mmHg or higher, a diastolic blood pressure of 90 mmHg or lower, or the use of antihypertensive medications within a span of two weeks [1]. The rise in obesity rates among the Indian population has contributed to an increase in hypertension cases, with projections indicating that one-third of the global population will be affected by hypertension within the next decade. Hypertension stands out as one of the primary risk factors for cardiovascular disease worldwide, and it is closely linked to a higher likelihood of death and disability [2]. Obesity exacerbates the situation by diminishing individuals' metabolic capacity and triggering various underlying health conditions, thus amplifying the risks associated with hypertension in terms of disability and mortality [3]. It is well-documented that high blood pressure often accompanies obesity, and the complex mechanisms linking fat accumulation to hypertension involve disruptions in renal function, insulin resistance, inflammation, and increased activity of the sympathetic nervous system [4]. The presence of hypertension significantly heightens the chances of experiencing severe health complications such as stroke, heart attack, and kidney disease [5]. Hence, the objective of this research is to identify a more efficient indicator for predicting the occurrence of hypertension. Factors related to population and social development, such as age and gender, have a significant impact on the prevalence of hypertension in India due to obesity [6]. The consumption of tobacco and alcohol has a strong correlation with the development of hypertension.

Conversely, various studies have demonstrated that engaging in regular vigorous physical activity is effective in preventing hypertension. It is crucial to assess the blood pressure and lipid profile of individuals, regardless of their obesity status, to accurately evaluate the risk, prevent cardiovascular diseases, and provide appropriate treatment [7]. Implementing strategies for weight management, lifestyle changes, and medication can help mitigate the negative effects of dyslipidemia and hypertension, ultimately reducing the overall cardiovascular risk [8]. The lipid profile and blood pressure are closely linked to obesity and play crucial roles in maintaining cardiovascular health [9]. By analyzing these indicators in both obese and non-obese individuals, healthcare professionals can tailor interventions to effectively reduce cardiovascular risk and enhance long-term outcomes [10]. Therefore, it is of interest to assess the significance of understanding lipid profile and blood pressure in the context of obesity for assessing and managing cardiovascular risk.

## Materials and Methods:

Throughout a span of one year, researchers conducted a cross-sectional study at a Tertiary Care Teaching Hospital to investigate the lipid profile and blood pressure of both obese and non-obese individuals. The study included participants aged between 30-60 years, categorized as non-obese (BMI < 30 kg/m^2^) or obese (BMI > 30 kg/m^2^). Patients with a history of diabetes, cardiovascular diseases, or other chronic conditions that could impact lipid metabolism or blood pressure were excluded from the study. Pregnant or lactating women, as well as individuals taking medications that could influence lipid profile or blood pressure, were also not included in the study.

## Inclusion criteria:

The study sample included all patients who suffer from obesity (BMI > 25 kg/m^2^ or obesity I and obesity II), adults over 25 years, and willing to give written permission after being given informed consent to take part in this research.

## Exclusion criteria:

History of diabetes mellitus, tuberculosis, heart, liver, or renal diseases or being pregnant/lactating at the time of research is collected. Additionally, immuno compromised patients and those with a history of hypersensitivity to the study medicines are also excluded.

## Data collection:

To determine BMI, individuals' height and weight are assessed using precise tools such as a stadiometer and scale. Blood pressure is measured utilizing a standardized method with an automated sphygmomanometer. These measurements are conducted while the individual is seated following a 5-minute relaxation period, ensuring accuracy by taking at least two readings and averaging them. Additionally, participants provide blood samples after fasting to assess their lipid profile. Furthermore, participants are required to complete a survey, providing details about their demographics, medical background, lifestyle habits, and medication usage.

## Data analysis:

Statistical summary was employed to describe the characteristics of the research sample, involving frequency distributions for qualitative data and mean ± standard deviation values for continuous variables. To compare variables between non-obese and obese groups, independent t-tests or non-parametric tests were conducted for blood pressure and lipid profiles. Chi-square tests were utilized for analyzing categorical variables. Additionally, a multivariable regression analysis was utilized to assess the association between obesity status, lipid profile parameters, blood pressure (dependent variables), while accounting for potential confounding variables like age, gender, and lifestyle habits.

## Results:

The research involved a total of 200 participants, with 100 classified as obese and 100 as non-obese. The average age of the non-obese participants was around 45.1±5.8 years, while the obese group had an average age of 43.2±6.3 years, indicating that the obese participants tended to be slightly older. The baseline characteristics for both obese and non-obese individuals are detailed in Table 1. There were a higher percentage of females in the obese group compared to the non-obese group (56% verses 42%). Alcohol and tobacco use were identified as significant risk factors for obesity, with obese individuals being much more likely to consume alcohol and smoke compared to non-obese individuals (obese versus non obese: OR: 12.3, 95% CI: 6.3-27.2; p: 0.0001 and OR: 45; 95%CI: 19.7-102.9; p: 0.001). Physical activity was strongly associated with the non-obese group, with those individuals more likely to engage in higher levels of physical activity (OR: 0.26; 95% CI: 0.1-0.6), p<0.002).

**Figure 1:**
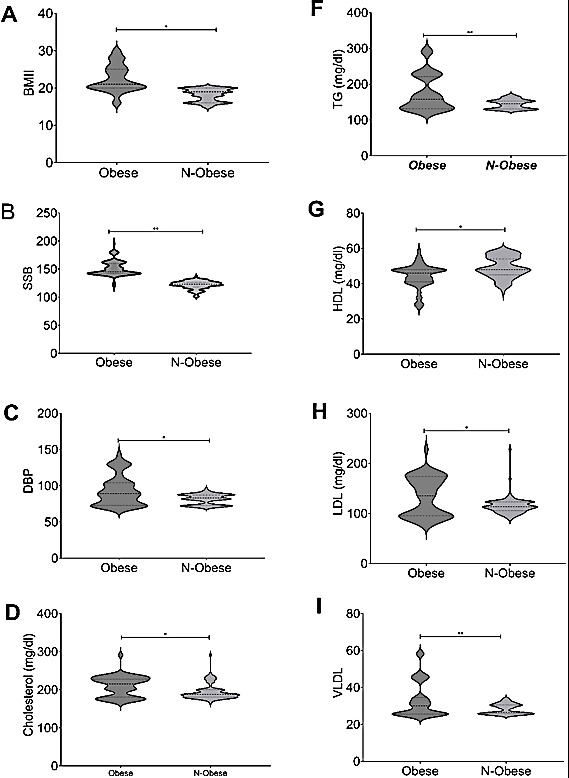
Distribution of BMI (A), BP (B-C) and lipid profile (D-I) in obese and non-obese subjects. *: p<0.05; **: p<0.01

Analysis of Body Mass Index (BMI) and Blood pressure readings demonstrated a clear distinction between individuals in the obese group and the non-obese group. The obese group exhibited markedly higher systolic and diastolic blood pressure levels compared to their non-obese counterparts, as demonstrated in Figure 1A. Specifically, the systolic blood pressure (SBP) for individuals in the non-obese group averaged at 121.7 mm Hg, while those in the obese group had a significantly elevated SBP of 151.2 mm Hg. Furthermore, the average diastolic blood pressure (DBP) for individuals in the obese group was notably higher at 93.2 mm Hg compared to those in the non-obese group, as illustrated in Figure 1B and Figure 1C. Additionally, the obese group exhibited significantly higher total cholesterol levels (209.3 mg/dL) in comparison to the non-obese group (193.7 mg/dL), as illustrated in Figure 1D. Thus, the higher triglyceride levels (318 mg/dL) in comparison to the non-obese group (150 mg/dL), as illustrated in Figure 1F.

On the other hand, there was a noticeable inverse correlation between HDL cholesterol levels and obesity, with the obese group showing lower levels (44.1 mg/dL) compared to the non-obese group (49.1 mg/dL) Figure 1G. Overall, The LDL cholesterol levels were also elevated in the obese group (137.4 mg/dL) compared to the non-obese group (121.3 mg/dL), as illustrated in Figure 1H., The VLDL cholesterol levels were also elevated in the obese group (65.8 mg/dL) compared to the non-obese group (36.3 mg/dL), as illustrated in Figure 1H., these findings underscore the substantial differences in baseline characteristics, lifestyle factors, and health markers between obese and non-obese individuals.

The results of the investigation indicated a favorable connection between total cholesterol (TC) and BMI (r = 0.331, p < 0.001), with TC levels tending to increase as BMI increased (r = 0.423, p < 0.001) (Table 2).

Additionally, there was a significant positive correlation between triglycerides (TG) and BMI, showing that BMI had a greater impact on TG levels. The study also found a positive association between LDL cholesterol and BMI, suggesting a potential link between higher LDL cholesterol levels and elevated BMI. Furthermore, multiple regression of this study demonstrated that for each unit increase in BMI, there was a predicted increase of 1.39 mm Hg in systolic blood pressure (SBP) when age and gender remained constant (Table 3).

## Discussion:

The reduction in HDL-C levels can be associated with several factors such as insulin resistance, inflammation, and changes in lipoprotein metabolism [11]. Non-obese individuals typically exhibit a healthier lipid profile compared to their obese counterparts [12]. It is important to note that lipid abnormalities can still occur in non-obese individuals due to genetic factors, dietary habits, physical activity levels, and other lifestyle factors [13]. Excess body fat can lead to the release of inflammatory substances that disrupt vascular function and increase peripheral resistance, ultimately leading to higher blood pressure [14]. Obesity, genetic factors, and lifestyle choices can all contribute to an increased risk of developing high blood pressure. However, even those who are not obese can still experience hypertension due to genetic predispositions, poor dietary habits, lack of physical activity, stress, and other lifestyle factors [15]. Some individuals who are not obese may also exhibit symptoms of metabolic syndrome, such as insulin resistance, dyslipidemia, hypertension, and abdominal obesity, which can further elevate their risk of cardiovascular issues [16]. Healthcare providers should regularly monitor the lipid profiles and blood pressure of both obese and non-obese individuals to assess their cardiovascular risk. Detecting dyslipidemia and hypertension early on allows for prompt interventions to reduce the risk of long-term complications [3]. In some cases, medications may be necessary to effectively manage dyslipidemia and hypertension [18]. Treatment decisions are made based on the patient's risk factors, existing medical conditions, and response to lifestyle changes. The field of personalized medicine has seen advancements in genetic testing and biomarker profiling, offering the potential to customize interventions to suit the unique characteristics of each patient and improve cardiovascular outcomes [19]. Innovative therapies that target specific genetic factors and metabolic profiles have the potential to transform the treatment of dyslipidemia, hypertension, and other cardiovascular risk factors [20]. It is crucial for public health efforts to focus on addressing obesity, promoting healthy lifestyles, and enhancing access to preventive care to lessen the impact of cardiovascular disease on a population level [21]. To improve cardiovascular health outcomes worldwide, a collaborative approach involving healthcare systems, policymakers, communities, and individuals is necessary to implement lasting changes that will benefit everyone [22].

## Conclusion:

The critical role of lipid profiles and blood pressure measurements in evaluating cardiovascular risk among both obese and non-obese individuals is reported. The findings reveal that while abnormalities in lipid levels and elevated blood pressure are prevalent in both groups, the patterns and implications of these abnormalities differ. Obese individuals are more likely to exhibit pronounced dyslipidemia and hypertension, contributing to a higher cardiovascular risk profile. However, non-obese individuals with elevated lipid levels and blood pressure are also at significant risk, highlighting the importance of comprehensive risk assessments that go beyond BMI alone. The research emphasizes the need for personalized prevention strategies that consider the unique risk factors of everyone. By doing so, healthcare providers can better identify high-risk individuals and implement targeted interventions to mitigate cardiovascular disease risk. Ultimately, this approach aims to enhance the effectiveness of primary prevention efforts, reducing the burden of cardiovascular diseases across diverse populations.

## Figures and Tables

**Table 1:** Baseline parameters of this study

**Parameters**	**Obese**	**Non-obese**	**OR (95% CI); p value**
Age (Mean±SD)	43.2±6.3	45.16±5.8	0.021
Gender			
Female	56	42	1.8 (1-3.1), 0.048*
Male	44	58	
Alcohol consumption			
Yes (more than one in month)	73	18	12.3 (6.3-27.2), 0.0001*
No	27	82	
Tobacco/smoking			
Current	86	12	45 (19.7-102.9), 0.0001*
No/ Former	14	88	
Taking activity			
Yes	8	25	0.26 (0.1-0.6), 0.002*
No	92	75	
OR: Odds Ratio; CI: Confidence Interval; probability; *: P< 0.05.

**Table 2:** Correlation between BMI & lipid profile parameters

**Lipid Profile Parameter**	**Pearson Correlation (r)**	**p-value**
Total cholesterol	0.331	<0.001
Triglycerides	0.423	<0.001
LDL-C	0.301	<0.001
HDL-C	-0.203	<0.001

**Table 3:** Multiple Regression Analysis for Blood pressure (BP) Levels

**Variable**	**Coefficient (β)**	**95% CI**	**p-value**
BMI	1.39	[1.21, 1.62]	<0.001
Age	0.03	[0.01, 0.08]	0.013
Gender (Male)	5.53	[4.01, 7.29]	<0.001
